# 1,1′-[(2,3,3a,4,5,6,7,7a-Octa­hydro-1*H*-1,3-benzimidazole-1,3-di­yl)bis­(methyl­ene)]bis­(1*H*-benzotriazole)

**DOI:** 10.1107/S160053681104044X

**Published:** 2011-10-29

**Authors:** Augusto Rivera, Dency José Pacheco, Jaime Ríos-Motta, Michaela Pojarová, Michal Dušek

**Affiliations:** a Departamento de Química, Universidad Nacional de Colombia, Ciudad Universitaria, Bogotá, Colombia; b Institute of Physics, AS CR, v.v.i., Na Slovance 2, 182 21 Praha 8, Czech Republic

## Abstract

The cyclo­hexane ring in the title compound, C_21_H_24_N_8_, adopts a chair conformation and the five-membered heterocyclic ring to which it is fused adopts a twist conformation on their common C—C bond. The substituents on the N atoms of the central five-membered heterocycle are arranged *trans* with respect to the central ring. The terminal benzotriazole rings are oriented at angles of 74.66 (8) and 84.18 (8)° with respect to the mean plane of the central heterocycle. The angle between the two benzotriazole rings is 30.80 (9)°. The bond lengths and angles are within normal ranges; the largest deviation from expecta­tion is for a long N—CH_2_ bond length [1.476 (2) Å] as a consequence of an anomeric effect. In the crystal, mol­ecules are connected by C—H⋯N hydrogen bonds.

## Related literature

For general background to anomeric effects, see: Carey & Sundberg (2000 ▶). For related structures see: Rivera *et al.* (2011 ▶); Wang *et al.* (2008 ▶). For ring conformations, see: Cremer & Pople (1975 ▶). For standard bond lengths, see: Allen *et al.* (1987 ▶).
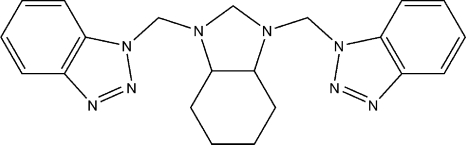


## Experimental

### 

#### Crystal data

C_21_H_24_N_8_
*M* *_r_* = 388.48
Monoclinic, 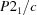

*a* = 11.9474 (2) Å
*b* = 5.9406 (1) Å
*c* = 27.3861 (4) Å
β = 90.861 (1)°
*V* = 1943.50 (5) Å^3^
*Z* = 4
Cu *K*α radiation
μ = 0.68 mm^−1^
*T* = 120 K
0.31 × 0.18 × 0.11 mm

#### Data collection

Oxford Diffraction Xcalibur Atlas Gemini ultra diffractometer
37999 measured reflections
3461 independent reflections
2990 reflections with *I* > 2σ(*I*)
*R*_int_ = 0.172

#### Refinement

*R*[*F* ^2^ > 2σ(*F* ^2^)] = 0.052
*wR*(*F* ^2^) = 0.137
*S* = 1.06
3461 reflections
262 parameters
H-atom parameters constrained
Δρ_max_ = 0.23 e Å^−3^
Δρ_min_ = −0.31 e Å^−3^

### 

Data collection: *CrysAlis PRO* (Oxford Diffraction, 2010 ▶); cell refinement: *CrysAlis PRO*; data reduction: *CrysAlis PRO*; program(s) used to solve structure: *SHELXS97* (Sheldrick, 2008 ▶); program(s) used to refine structure: *SHELXL97* (Sheldrick, 2008 ▶); molecular graphics: *DIAMOND* (Brandenburg & Putz, 2005 ▶); software used to prepare material for publication: *publCIF* (Westrip, 2010 ▶).

## Supplementary Material

Crystal structure: contains datablock(s) I, global. DOI: 10.1107/S160053681104044X/bh2381sup1.cif

Structure factors: contains datablock(s) I. DOI: 10.1107/S160053681104044X/bh2381Isup2.hkl

Supplementary material file. DOI: 10.1107/S160053681104044X/bh2381Isup3.cml

Additional supplementary materials:  crystallographic information; 3D view; checkCIF report

## Figures and Tables

**Table 1:** Hydrogen-bond geometry (Å, °)

*D*—H⋯*A*	*D*—H	H⋯*A*	*D*⋯*A*	*D*—H⋯*A*
C7—H7b⋯N1^i^	0.97	2.38	3.301 (2)	159
C15—H15b⋯N7^ii^	0.97	2.62	3.504 (2)	151

Symmetry codes: (i) 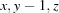; (ii) 
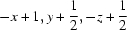
.

**Fig. 1.**
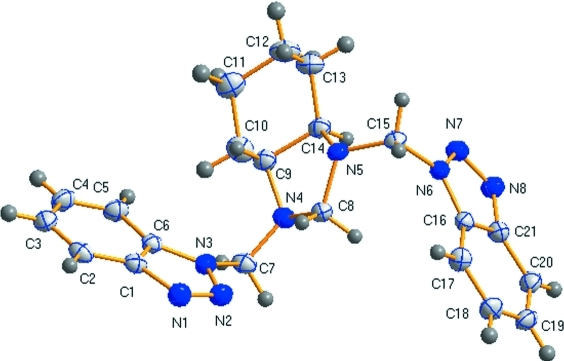
A view of the title compound, with displacement ellipsoids drawn at the 50% probability level.

**Fig. 2.**
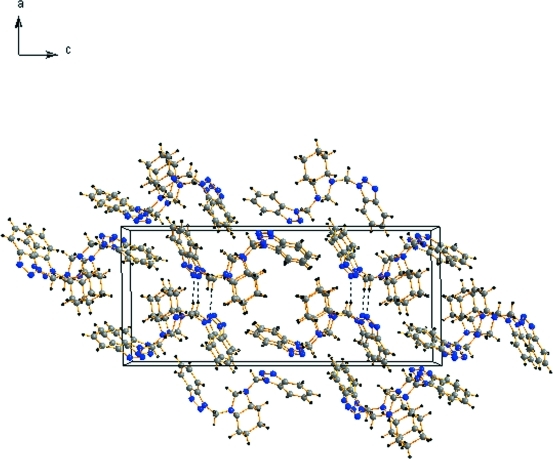
Crystal packing of the title compound viewed along b axis.

## supplementary crystallographic information

### Comment

Among the most thoroughly studied stereoelectronic effects, the interactions between lone pairs have attracted much interest. These interactions between a non bonded electron pair and antibonding *σ** sigma bonds usually play a feature role in the preferred conformations of such systems (Carey & Sundberg, 2000). The data of the crystal structure of the title compound indicate the occurrence of a *n*(N)→*σ*^*^(C—N) electron delocalization, characteristic of the anomeric effect, as evidenced by the lengthened bond N6—C15 [1.476 (2) Å], shortened bond C15—N5 [1.433 (2)] and distorted C—N—C bond angles, C8—N5—C14 = 106.11 (13)°, C8—N5—C15 = 115.53 (13)°, and C14—N5—C15 = 118.03 (14)°. These results exhibit the same pattern of C—N shortened bond lengths that the crystal structure of 1,3-bis[(1*H*-benzotriazol-1-yl)methyl]-2,3-dihydro-1*H*-benzimidazole recently reported (Rivera *et al.*, 2011). The structural parameters suggest an increase in *p*-character of nitrogen and reduction in N-pyramidality. The title compound was obtained by the reaction of racemic cyclic aminal (2*R*,7*R*,11*S*,16*S*)-1,8,10,17-tetraazapentacyclo-[8.8.1.1^8,17^.0^2,7^.0^11,16^]icosane with two equivalents of benzotriazole. The molecular structure and atom-numbering scheme are shown in Fig. 1. The asymmetric unit comprises of one molecule of the title compound (Fig 1). The bond lengths (Allen *et al.*, 1987) and angles are generally within normal ranges (Wang *et al.*, 2008).

The cyclohexane ring adopts a chair conformation and the five-membered ring to which it is fused adopts a twist conformation on C9—C14 with Q(2) = 0.455 (2) Å and φ = 311.1 (2)° (Cremer & Pople, 1975) and a tr*ans* disubstitution. The benzotriazole rings (N1—N3/C1—C6; N6—N8/C16—C21) are essentially planar with the maximum deviations from planarity being 0.0220 (19) Å for atom C3 and -0.0161 (19) for atom C18. The central heterocyclic ring makes an angle of 74.66 (8)° and 84.18 (8)° with the planar benzotriazole rings. The angle between the two benzotriazole rings is 30.80 (9)°. The two exocyclic bonds of methylene carbon atoms occupy pseudo-axial positions.

The crystal structure contains an intermolecular C7—H7B···N1 hydrogen bond between one H atom of the N—CH_2_—N group (aminal group) and one N atom of the benzotriazole ring of neighboring molecule linking adjacent molecules to form a one-dimensional chain running parallel to the *b* axis (Fig. 2), and further linked by weak C15—H15B···N intermolecular interactions. The distance of 3.4502 (9) Å between the centroids of the rings N1/N2/N3/C6/C1 related by the symmetry code (-*x*, 2 - *y*, -*z*) suggests a possible π–π interaction in the crystal.

### Experimental

A solution of (2*R*,7*R*,11*S*,16*S*)-1,8,10,17-tetraazapentacyclo-[8.8.1.1^8,17^.0^2,7^.0^11,16^]icosane (276 mg, 1.00 mmol) in dioxane (3 ml) and water (4 ml), previously prepared following described procedures, was added dropwise in a dioxane solution (3 ml) containing two equivalents of benzotriazole (238 mg, 2.00 mmol) in a two-necked round-bottomed flask. The mixture was stirred for about 6 h. and then the solvent was evaporated under reduced pressure until a sticky residue appeared. The product was purified by chromatography on a silica column, and subjected to gradient elution with benzene:ethyl acetate (yield 75%, m.p. = 424–425 K). Single crystals of the racemic title compound were grown from a chloroform solution by slow evaporation of the solvent at room temperature over a period of about 2 weeks.

### Refinement

The quality of the crystals was very low. The selected crystal for measurement was the best one from several attempts. All H atoms were added in calculated positions and refined as riding with C–H distances of 0.93 or 0.97 Å. The isotropic atomic displacement parameters of H atoms were fixed to 1.2×*U*_eq_ of the parent atom.

### Crystal data

**Table:**

C_21_H_24_N_8_	*F*(000) = 824
*M**_r_* = 388.48	*D*_x_ = 1.328 Mg m^−^^3^
Monoclinic, *P*2_1_/*c*	Melting point: 424 K
Hall symbol: -P 2ybc	Cu *K*α radiation, λ = 1.5418 Å
*a* = 11.9474 (2) Å	Cell parameters from 13493 reflections
*b* = 5.9406 (1) Å	θ = 3.2°
*c* = 27.3861 (4) Å	µ = 0.68 mm^−^^1^
β = 90.861 (1)°	*T* = 120 K
*V* = 1943.50 (5) Å^3^	Plate, colourless
*Z* = 4	0.31 × 0.18 × 0.11 mm

### Data collection

**Table:**

Oxford Diffraction Xcalibur Atlas Gemini ultra diffractometer	2990 reflections with *I* > 2σ(*I*)
Radiation source: Enhance Ultra (Cu) X-ray Source	*R*_int_ = 0.172
mirror	θ_max_ = 67.1°, θ_min_ = 3.2°
Detector resolution: 10.3784 pixels mm^-1^	*h* = −14→14
Rotation method data acquisition using ω scans	*k* = −7→7
37999 measured reflections	*l* = −32→32
3461 independent reflections	

### Refinement

**Table:**

Refinement on *F*^2^	Primary atom site location: structure-invariant direct methods
Least-squares matrix: full	Secondary atom site location: difference Fourier map
*R*[*F*^2^ > 2σ(*F*^2^)] = 0.052	Hydrogen site location: inferred from neighbouring sites
*wR*(*F*^2^) = 0.137	H-atom parameters constrained
*S* = 1.06	*w* = 1/[σ^2^(*F*_o_^2^) + (0.0756*P*)^2^ + 0.4705*P*] where *P* = (*F*_o_^2^ + 2*F*_c_^2^)/3
3461 reflections	(Δ/σ)_max_ < 0.001
262 parameters	Δρ_max_ = 0.23 e Å^−^^3^
0 restraints	Δρ_min_ = −0.31 e Å^−^^3^
0 constraints	

### Fractional atomic coordinates and isotropic or equivalent isotropic displacement parameters (Å^2^)

**Table:**

	*x*	*y*	*z*	*U*_iso_*/*U*_eq_	
N1	0.06576 (12)	1.3038 (2)	0.03014 (5)	0.0301 (4)	
N2	0.06561 (12)	1.1599 (2)	0.06570 (5)	0.0298 (3)	
N3	0.12624 (11)	0.9755 (2)	0.05292 (5)	0.0256 (3)	
N4	0.21777 (12)	0.7711 (2)	0.12114 (5)	0.0277 (3)	
N5	0.34776 (11)	0.9703 (2)	0.16832 (5)	0.0250 (3)	
N6	0.31150 (11)	0.9133 (2)	0.25550 (5)	0.0240 (3)	
N7	0.34948 (11)	0.7097 (3)	0.27089 (5)	0.0296 (4)	
N8	0.27753 (12)	0.6222 (3)	0.30094 (5)	0.0302 (3)	
C1	0.12747 (13)	1.2136 (3)	−0.00732 (6)	0.0249 (4)	
C2	0.14940 (14)	1.3000 (3)	−0.05407 (6)	0.0293 (4)	
H2	0.1233	1.4406	−0.0639	0.035*	
C3	0.21134 (15)	1.1660 (3)	−0.08439 (7)	0.0332 (4)	
H3	0.2266	1.2157	−0.1158	0.040*	
C4	0.25227 (15)	0.9548 (3)	−0.06892 (7)	0.0334 (4)	
H4	0.2949	0.8705	−0.0904	0.040*	
C5	0.23166 (14)	0.8690 (3)	−0.02351 (7)	0.0299 (4)	
H5	0.2591	0.7296	−0.0136	0.036*	
C6	0.16690 (13)	1.0034 (3)	0.00695 (6)	0.0239 (4)	
C7	0.12758 (14)	0.7809 (3)	0.08616 (7)	0.0288 (4)	
H7A	0.0577	0.7797	0.1038	0.035*	
H7B	0.1296	0.6450	0.0666	0.035*	
C8	0.22853 (13)	0.9669 (3)	0.15340 (6)	0.0270 (4)	
H8A	0.1810	0.9509	0.1816	0.032*	
H8B	0.2083	1.1041	0.1362	0.032*	
C9	0.33087 (14)	0.7201 (3)	0.10497 (6)	0.0265 (4)	
H9	0.3541	0.8339	0.0813	0.032*	
C10	0.35246 (16)	0.4874 (3)	0.08427 (7)	0.0350 (4)	
H10A	0.3126	0.4691	0.0534	0.042*	
H10B	0.3265	0.3730	0.1067	0.042*	
C11	0.47850 (16)	0.4632 (3)	0.07680 (8)	0.0391 (5)	
H11A	0.5017	0.5670	0.0515	0.047*	
H11B	0.4945	0.3117	0.0657	0.047*	
C12	0.54586 (16)	0.5107 (3)	0.12368 (8)	0.0391 (5)	
H12A	0.6251	0.5020	0.1167	0.047*	
H12B	0.5292	0.3956	0.1476	0.047*	
C13	0.51985 (14)	0.7429 (3)	0.14530 (7)	0.0338 (4)	
H13A	0.5590	0.7621	0.1763	0.041*	
H13B	0.5437	0.8607	0.1233	0.041*	
C14	0.39463 (14)	0.7551 (3)	0.15242 (6)	0.0265 (4)	
H14	0.3728	0.6377	0.1755	0.032*	
C15	0.37030 (13)	1.0415 (3)	0.21750 (6)	0.0270 (4)	
H15A	0.3498	1.1989	0.2204	0.032*	
H15B	0.4502	1.0302	0.2238	0.032*	
C16	0.21029 (13)	0.9579 (3)	0.27606 (6)	0.0235 (4)	
C17	0.13513 (14)	1.1384 (3)	0.27189 (6)	0.0284 (4)	
H17	0.1497	1.2636	0.2526	0.034*	
C18	0.03858 (15)	1.1180 (3)	0.29813 (7)	0.0333 (4)	
H18	−0.0144	1.2324	0.2960	0.040*	
C19	0.01666 (14)	0.9313 (3)	0.32806 (6)	0.0313 (4)	
H19	−0.0497	0.9267	0.3454	0.038*	
C20	0.09064 (14)	0.7563 (3)	0.33234 (6)	0.0297 (4)	
H20	0.0762	0.6333	0.3523	0.036*	
C21	0.18967 (14)	0.7708 (3)	0.30514 (6)	0.0255 (4)	

### Atomic displacement parameters (Å^2^)

**Table:**

	*U*^11^	*U*^22^	*U*^33^	*U*^12^	*U*^13^	*U*^23^
N1	0.0331 (8)	0.0264 (8)	0.0309 (8)	0.0061 (6)	−0.0009 (6)	−0.0036 (6)
N2	0.0288 (7)	0.0288 (8)	0.0320 (8)	0.0062 (6)	0.0007 (6)	−0.0046 (6)
N3	0.0240 (7)	0.0252 (7)	0.0275 (8)	0.0008 (5)	0.0001 (6)	−0.0031 (6)
N4	0.0231 (7)	0.0284 (8)	0.0316 (8)	−0.0020 (5)	−0.0018 (6)	0.0023 (6)
N5	0.0199 (7)	0.0294 (8)	0.0258 (8)	−0.0033 (5)	0.0033 (5)	0.0013 (6)
N6	0.0203 (6)	0.0282 (7)	0.0233 (7)	−0.0007 (5)	0.0004 (5)	0.0026 (5)
N7	0.0238 (7)	0.0329 (8)	0.0320 (8)	0.0046 (6)	0.0004 (6)	0.0050 (6)
N8	0.0263 (7)	0.0314 (8)	0.0329 (8)	0.0032 (6)	0.0028 (6)	0.0057 (6)
C1	0.0228 (8)	0.0221 (8)	0.0298 (9)	−0.0012 (6)	−0.0019 (6)	−0.0047 (6)
C2	0.0281 (8)	0.0257 (9)	0.0338 (10)	−0.0039 (7)	−0.0051 (7)	0.0021 (7)
C3	0.0281 (9)	0.0409 (10)	0.0308 (9)	−0.0068 (7)	0.0025 (7)	0.0020 (8)
C4	0.0283 (9)	0.0375 (10)	0.0345 (10)	0.0027 (7)	0.0063 (7)	−0.0074 (8)
C5	0.0267 (8)	0.0271 (9)	0.0361 (10)	0.0033 (7)	0.0018 (7)	−0.0047 (7)
C6	0.0204 (7)	0.0258 (8)	0.0255 (9)	−0.0025 (6)	−0.0025 (6)	−0.0034 (6)
C7	0.0256 (8)	0.0261 (9)	0.0345 (10)	−0.0034 (6)	−0.0024 (7)	0.0021 (7)
C8	0.0218 (8)	0.0345 (9)	0.0247 (9)	−0.0010 (7)	0.0016 (6)	0.0004 (7)
C9	0.0249 (8)	0.0247 (9)	0.0299 (9)	−0.0019 (6)	0.0020 (7)	0.0035 (7)
C10	0.0373 (10)	0.0263 (9)	0.0412 (11)	0.0003 (7)	−0.0049 (8)	−0.0014 (8)
C11	0.0403 (11)	0.0298 (10)	0.0473 (12)	0.0066 (8)	0.0055 (9)	−0.0036 (8)
C12	0.0282 (9)	0.0362 (11)	0.0530 (13)	0.0040 (8)	0.0008 (8)	0.0004 (9)
C13	0.0230 (9)	0.0353 (10)	0.0431 (11)	−0.0004 (7)	0.0007 (7)	0.0002 (8)
C14	0.0236 (8)	0.0259 (9)	0.0299 (9)	−0.0024 (6)	0.0028 (7)	0.0046 (7)
C15	0.0223 (8)	0.0304 (9)	0.0283 (9)	−0.0073 (6)	0.0021 (6)	0.0022 (7)
C16	0.0204 (8)	0.0276 (9)	0.0225 (8)	−0.0011 (6)	−0.0003 (6)	−0.0027 (6)
C17	0.0319 (9)	0.0243 (9)	0.0291 (9)	0.0023 (7)	0.0012 (7)	−0.0005 (7)
C18	0.0324 (9)	0.0346 (10)	0.0330 (10)	0.0088 (7)	0.0013 (7)	−0.0074 (7)
C19	0.0252 (8)	0.0393 (10)	0.0297 (10)	−0.0011 (7)	0.0067 (7)	−0.0085 (7)
C20	0.0284 (9)	0.0333 (10)	0.0274 (9)	−0.0055 (7)	0.0037 (7)	0.0010 (7)
C21	0.0247 (8)	0.0268 (9)	0.0250 (9)	0.0005 (6)	0.0006 (6)	0.0004 (6)

### Geometric parameters (Å, °)

**Table:**

N1—N2	1.296 (2)	C8—H8B	0.9700
N1—C1	1.380 (2)	C9—C14	1.511 (2)
N2—N3	1.3619 (19)	C9—C10	1.517 (2)
N3—C6	1.366 (2)	C9—H9	0.9800
N3—C7	1.471 (2)	C10—C11	1.529 (3)
N4—C7	1.432 (2)	C10—H10A	0.9700
N4—C9	1.460 (2)	C10—H10B	0.9700
N4—C8	1.465 (2)	C11—C12	1.531 (3)
N5—C15	1.433 (2)	C11—H11A	0.9700
N5—C14	1.464 (2)	C11—H11B	0.9700
N5—C8	1.476 (2)	C12—C13	1.535 (3)
N6—N7	1.357 (2)	C12—H12A	0.9700
N6—C16	1.367 (2)	C12—H12B	0.9700
N6—C15	1.476 (2)	C13—C14	1.513 (2)
N7—N8	1.307 (2)	C13—H13A	0.9700
N8—C21	1.378 (2)	C13—H13B	0.9700
C1—C6	1.389 (2)	C14—H14	0.9800
C1—C2	1.408 (2)	C15—H15A	0.9700
C2—C3	1.375 (3)	C15—H15B	0.9700
C2—H2	0.9300	C16—C21	1.391 (2)
C3—C4	1.409 (3)	C16—C17	1.402 (2)
C3—H3	0.9300	C17—C18	1.374 (2)
C4—C5	1.370 (3)	C17—H17	0.9300
C4—H4	0.9300	C18—C19	1.406 (3)
C5—C6	1.397 (2)	C18—H18	0.9300
C5—H5	0.9300	C19—C20	1.369 (3)
C7—H7A	0.9700	C19—H19	0.9300
C7—H7B	0.9700	C20—C21	1.410 (2)
C8—H8A	0.9700	C20—H20	0.9300
			
N2—N1—C1	108.00 (14)	C9—C10—H10A	110.1
N1—N2—N3	109.32 (13)	C11—C10—H10A	110.1
N2—N3—C6	109.70 (13)	C9—C10—H10B	110.1
N2—N3—C7	118.30 (13)	C11—C10—H10B	110.1
C6—N3—C7	131.64 (14)	H10A—C10—H10B	108.4
C7—N4—C9	119.73 (14)	C10—C11—C12	112.13 (16)
C7—N4—C8	115.44 (13)	C10—C11—H11A	109.2
C9—N4—C8	105.94 (13)	C12—C11—H11A	109.2
C15—N5—C14	118.03 (14)	C10—C11—H11B	109.2
C15—N5—C8	115.53 (13)	C12—C11—H11B	109.2
C14—N5—C8	106.11 (13)	H11A—C11—H11B	107.9
N7—N6—C16	109.79 (13)	C11—C12—C13	112.45 (16)
N7—N6—C15	121.24 (13)	C11—C12—H12A	109.1
C16—N6—C15	128.57 (14)	C13—C12—H12A	109.1
N8—N7—N6	109.28 (13)	C11—C12—H12B	109.1
N7—N8—C21	107.87 (14)	C13—C12—H12B	109.1
N1—C1—C6	108.83 (15)	H12A—C12—H12B	107.8
N1—C1—C2	130.01 (16)	C14—C13—C12	107.41 (14)
C6—C1—C2	121.12 (16)	C14—C13—H13A	110.2
C3—C2—C1	116.57 (16)	C12—C13—H13A	110.2
C3—C2—H2	121.7	C14—C13—H13B	110.2
C1—C2—H2	121.7	C12—C13—H13B	110.2
C2—C3—C4	121.44 (17)	H13A—C13—H13B	108.5
C2—C3—H3	119.3	N5—C14—C9	100.73 (13)
C4—C3—H3	119.3	N5—C14—C13	117.62 (14)
C5—C4—C3	122.54 (17)	C9—C14—C13	111.67 (14)
C5—C4—H4	118.7	N5—C14—H14	108.8
C3—C4—H4	118.7	C9—C14—H14	108.8
C4—C5—C6	115.99 (16)	C13—C14—H14	108.8
C4—C5—H5	122.0	N5—C15—N6	115.18 (13)
C6—C5—H5	122.0	N5—C15—H15A	108.5
N3—C6—C1	104.14 (14)	N6—C15—H15A	108.5
N3—C6—C5	133.54 (16)	N5—C15—H15B	108.5
C1—C6—C5	122.31 (16)	N6—C15—H15B	108.5
N4—C7—N3	116.54 (13)	H15A—C15—H15B	107.5
N4—C7—H7A	108.2	N6—C16—C21	104.25 (14)
N3—C7—H7A	108.2	N6—C16—C17	133.15 (15)
N4—C7—H7B	108.2	C21—C16—C17	122.59 (15)
N3—C7—H7B	108.2	C18—C17—C16	115.60 (16)
H7A—C7—H7B	107.3	C18—C17—H17	122.2
N4—C8—N5	104.68 (13)	C16—C17—H17	122.2
N4—C8—H8A	110.8	C17—C18—C19	122.65 (16)
N5—C8—H8A	110.8	C17—C18—H18	118.7
N4—C8—H8B	110.8	C19—C18—H18	118.7
N5—C8—H8B	110.8	C20—C19—C18	121.58 (16)
H8A—C8—H8B	108.9	C20—C19—H19	119.2
N4—C9—C14	99.64 (13)	C18—C19—H19	119.2
N4—C9—C10	117.77 (14)	C19—C20—C21	117.01 (16)
C14—C9—C10	111.14 (14)	C19—C20—H20	121.5
N4—C9—H9	109.3	C21—C20—H20	121.5
C14—C9—H9	109.3	N8—C21—C16	108.80 (14)
C10—C9—H9	109.3	N8—C21—C20	130.64 (16)
C9—C10—C11	107.98 (15)	C16—C21—C20	120.56 (15)
			
N3—C7—N4—C8	56.8 (4)	C8—N5—C15—N6	55.5 (4)

### Hydrogen-bond geometry (Å, °)

**Table:**

*D*—H···*A*	*D*—H	H···*A*	*D*···*A*	*D*—H···*A*
C8—H8a···N6	0.97	2.55	2.970 (2)	106
C8—H8b···N3	0.97	2.58	2.995 (2)	106
C7—H7b···N1^i^	0.97	2.38	3.301 (2)	159
C15—H15b···N7^ii^	0.97	2.62	3.504 (2)	151

Symmetry codes: (i) *x*, *y*−1, *z*; (ii) −*x*+1, *y*+1/2, −*z*+1/2.
